# Short-term differential adaptation to anaerobic stress via genomic mutations by *Escherichia coli* strains K-12 and B lacking alcohol dehydrogenase

**DOI:** 10.3389/fmicb.2014.00476

**Published:** 2014-09-09

**Authors:** Hyun Ju Kim, Haeyoung Jeong, Seungwoo Hwang, Moo-Seung Lee, Yong-Jik Lee, Dong-Woo Lee, Sang Jun Lee

**Affiliations:** ^1^Biosystems and Bioengineering Program, University of Science and Technology (UST)Daejeon, South Korea; ^2^Infection and Immunity Research Center, Korea Research Institute of Bioscience & Biotechnology (KRIBB)Daejeon, South Korea; ^3^Korean Bioinformation Center, Korea Research Institute of Bioscience & Biotechnology (KRIBB)Daejeon, South Korea; ^4^School of Applied Biosciences, Kyungpook National UniversityDaegu, South Korea

**Keywords:** anaerobic condition, alcohol dehydrogenase, adaptation, genomic mutation, *pta* gene, *pflB* gene, redox balance

## Abstract

Microbial adaptations often occur via genomic mutations under adverse environmental conditions. This study used *Escherichia coli* Δ*adhE* cells as a model system to investigate adaptation to anaerobic conditions, which we then compared with the adaptive mechanisms of two closely related *E. coli* strains, K-12 and B. In contrast to K-12 Δ*adhE* cells, the *E. coli* B Δ*adhE* cells exhibited significantly delayed adaptive growth under anaerobic conditions. Adaptation by the K-12 and B strains mainly employed anaerobic lactate fermentation to restore cellular growth. Several mutations were identified in the *pta* or *pflB* genes of adapted K-12 cells, but mostly in the *pta* gene of the B strains. However, the types of mutation in the adapted K-12 and B strains were similar. Cellular viability was affected directly by severe redox imbalance in B Δ*adhE* cells, which also impaired their ability to adapt to anaerobic conditions. This study demonstrates that closely related microorganisms may undergo different adaptations under the same set of adverse conditions, which might be associated with the specific metabolic characteristics of each strain. This study provides new insights into short-term microbial adaptation to stressful conditions, which may reflect dynamic microbial population changes in nature.

## Introduction

Stress has been defined as “any deviation from optimal growth conditions that results in a reduced growth rate” (Storz and Hengge-Aronis, 2000). Sources of bacterial stress include irradiation, heat shock, osmotic pressure, pH changes, starvation, oxygen radicals, aerobic to anaerobic transition, and oxygen deprivation (Patschkowski et al., 2000; Moat et al., 2002b). In addition, imbalances in intracellular metabolites, such as sugar phosphate accumulation (Lee et al., 2014), nucleotide depletion (Lee et al., 2009), and elevated NADPH levels (Auriol et al., 2011) due to the absence of relevant metabolic enzymes, can cause retarded cellular growth, which are also forms of stress.

Microorganisms have evolved a variety of alternative and/or bypass pathways to maintain their metabolic functionality in response to different environmental conditions. Under unfavorable growth conditions, microbes can sustain their viability by activating various biochemical pathways to maintain homeostasis (or the cellular growth rate), where their metabolism may be regulated by two-component systems (Lynch and Lin, 1996; Bekker et al., 2006), sigma factors (Battesti et al., 2011), regulator proteolysis (Mettert and Kiley, 2005; Mika and Hengge, 2005), small RNAs (Durand and Storz, 2010), alarmones (Mechold et al., 2013), and other mechanisms. To cope with severe environmental stress, adaptation can emerge via the acquisition of random mutations, which may be positively selected in a population if the mutations generate a beneficial phenotype (Rando and Verstrepen, 2007).

Recent studies show that mutations beneficial for optimal cell growth can be identified after long-term evolution and adaptation (Barrick et al., 2009; Conrad et al., 2010). Specific mutations such as *rpoC* mutations are found repeatedly after adaptation in minimal growth medium (Conrad et al., 2010), whereas diverse beneficial mutations can be detected in microbial populations that evolve at high temperature (Tenaillon et al., 2012). Efforts have been made to study the evolutionary pathways utilized under stress conditions (such as antibiotic resistance) using continuous culture (Toprak et al., 2012). Time course experiments may also be helpful for elucidating how stress affects microbial populations and when compensatory mutants arise during adaptation to adverse conditions.

**Figure 1:**
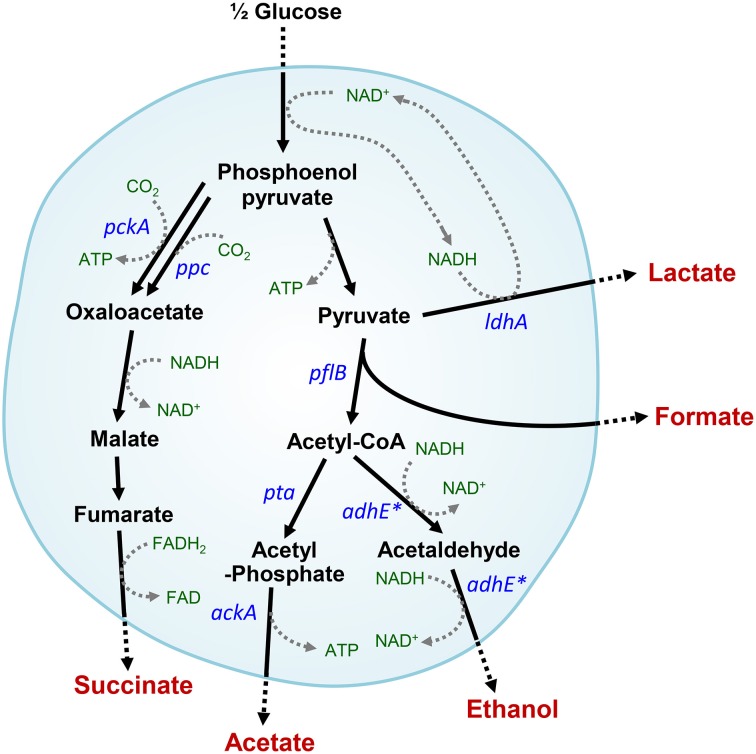
**Intracellular anaerobic NAD^+^ recycling in mixed acid fermentation by lactate dehydrogenase in Δ*adhE* mutant cells**. Metabolic pathways affected by the *adhE* mutation are indicated by asterisks.

Under anaerobic conditions, *Escherichia coli* convert glucose into formate, acetate, ethanol, lactate, and succinate (Clark, 1989; Moat et al., 2002a). The mixed acid fermentation is required to recycle redox cofactors, which can occur via several reactions catalyzed by alcohol or acid dehydrogenases in cells (Figure 1). Alteration or impairment of the redox balance caused by a lack of appropriate electron acceptors, carriers, or redox-related enzymes can result in stress in microbial cells (Gonzalez-Siso et al., 2009). In the absence of alcohol dehydrogenase (encoded by *adhE*), which plays a key role in the maintenance of redox balance via NADH oxidation (or NAD^+^ recycling) under anaerobic conditions, cells experience severe redox-stress during the transition from aerobic to anaerobic conditions (Gupta and Clark, 1989; Galinina et al., 2012). *E. coli* mutant cells that lack the *adhE* gene are unable to grow anaerobically, whereas additional *pta* (phosphotransacetylase) mutations enable *adhE* mutants to grow via lactate fermentation (Gupta and Clark, 1989) (Figure 1).

In our previous genome-wide analysis of the redox reactions that are important for anaerobic growth (Kim et al., 2013), we serendipitously found that *E. coli* K-12 Δ*adhE* smoothly grew via lactate fermentation in liquid culture under anaerobic conditions. By contrast, BL21(DE3) Δ*adhE* cells exhibited highly delayed anaerobic growth. When fermentation broths of K-12 Δ*adhE* and BL21(DE3) Δ*adhE* strains were respectively taken to regrow in the same fresh broth, growth rates of K-12 Δ*adhE* and BL21(DE3) Δ*adhE* strains were accelerated without any lag periods and comparable to that exhibited by the wild-type strain. The present study examined the different adaptations (genomic mutations and metabolic characteristics) of two closely related *E. coli* strains (K-12 and B) grown under the same stress conditions. This study provides insights into how microbial populations can change rapidly and adapt to adverse environmental conditions via genomic mutations, which might be a common feature of microbial outbreaks and population changes in nature.

## Materials and methods

### Bacterial strains

*E. coli* strains are listed in Table 1. The *E. coli* B (REL606) strain was kindly provided by Richard E. Lenski at Michigan State University. The *E. coli* B strains BL21 and BL21(DE3) were purchased from Promega (Madison, WI) and Invitrogen (Carlsbad, CA), respectively. The *E. coli* K-12 strains, MG1655, BW25113, and W3110 were kindly provided by Sankar Adhya at the NIH, the Coli Genetic Stock Center (CGSC) at Yale University, and Korean Collection for Type Cultures (KCTC) at KRIBB, respectively.

**Table 1:** **Bacterial strains, phages, and plasmids used in this study**.

**Name**	**Relevant genotypes or characteristics**	**Reference or source**
***E. COLI* K-12**		
BW25113	Δ*(araD-araB)567* Δ*lacZ4787(::rrnB-3)* λ^−^ *rph-1* Δ*(rhaD-rhaB)568 hsdR514*	CGSC
MG1655	F^−^ *ilvG rfb*-50 *rph*-1	S. Adhya
W3110	F^−^ λ^−^*rph-1* INV(*rrnD,rrnE*)	KCTC
JW1228^*^	BW25113 Δ*adhE*::FRT-KmR-FRT	Keio collection
JW0883	BW25113 Δ*ycaN*::FRT-KmR-FRT	Keio collection
JW2291	BW25113 Δ*yfbU*::FRT-KmR-FRT	Keio collection
JW1317	BW25113 Δ*tpx*::FRT-KmR-FRT, *fnr^+^*	Keio collection
JW0739	BW25113 Δ*galM*::FRT-KmR-FRT, *modE^+^*	Keio collection
JW0432	BW25113 Δ*ybaV*::FRT-KmR-FRT, *lon^+^*	Keio collection
JW0556	BW25113 Δ*ybcH*::FRT-KmR-FRT, *ompT^+^*	Keio collection
JW1378	BW25113 Δ*ydbK*::FRT-KmR-FRT, *recE^+^*	Keio collection
JW5793	BW25113 Δ*yjjN*::FRT-KmR-FRT, *hsd^K-12^*	Keio collection
JW4128	BW25113 Δ*mutL*::FRT-KmR-FRT	Keio collection
JW2703	BW25113 Δ*mutS*::FRT-KmR-FRT	Keio collection
JW0097	BW25113 Δ*mutT*::FRT-KmR-FRT	Keio collection
JW0886	BW25113 Δ*pflB*::FRT-KmR-FRT	Keio collection
JW2294	BW25113 Δ*pta*::FRT-KmR-FRT	Keio collection
HK108	BW25113 Δ*adhE*::FRT	This study
HK104	MG1655 Δ*adhE*::FRT-KmR-FRT	This study
HK256	W3110 Δ*adhE*::FRT-KmR-FRT	This study
HK185^*^	JW1228 *pta* (C1870A substitution)	This study
HK187	JW1228 *pta* (G1780A substitution)	This study
HK188^*^	JW1228 *pflB* (IS4 insertion at promoter)	This study
HK183	BW25113 Δ*adhE*::FRT *pflB* (G1105A)	This study
HK155	BW25113 Δ*adhE*::FRT *pta* (C2074T)	This study
HK414	BW25113 Δ*adhE*::FRT *pflB* (G1105A) Δ*ycaN*::KmR	This study
HK427	BW25113 Δ*adhE*::FRT *pta* (C2074T) Δ*yfbU*::KmR	This study
HK235	BW25113 Δ*adhE*::FRT Δ*pflB*::FRT-KmR-FRT	This study
HK227	BW25113 Δ*adhE*::FRT Δ*pta*::FRT-KmR-FRT	This study
***E. COLI* B**		
BL21(DE3)	F^−^ *ompT gal dcm lon hsdSB*(r^−^_B_ m^−^_B_) λ (DE3 [*lacI lacUV5*-T7 gene 1 *ind1 sam7 nin5*])	Invitrogen
BL21	F^−^ *dcm ompT hsdS*(r^−^_B_ m^−^_B_) *gal* [malB^+^]_K-12_(λ^S^)	Promega
REL606	F^−^ *lon mal^+^* λ^S^ T6^R^ *str*^R^*rm*_111_ *ara*^−^	Richard E. Lenski
HK105	BL21 Δ*adhE*::FRT-KmR-FRT	This study
HK106^*^	BL21(DE3) Δ*adhE*::FRT-KmR-FRT	This study
HK110	BL21(DE3) Δ*adhE*::FRT	This study
HK255	REL606 Δ*adhE*::FRT-KmR-FRT	This study
HK121^*^	HK110 *pta* (G219 deletion)	This study
HK122^*^	HK110 *pta* (A1967C substitution)	This study
HK201^*^	HK110 *pta* (T2018A substitution)	This study
HK501	BL21(DE3) Δ*adhE*::FRT *pflB* (G1931A)	This study
HK441	BL21(DE3) Δ*adhE*::FRT *pta* (A1967C) Δ*yfbU*::KmR	This study
HK547	BL21(DE3) Δ*adhE*::FRT *pflB* (G1931A) Δ*ycaN*::KmR	This study
HK236	BL21(DE3) Δ*adhE*::FRT Δ*pflB*::FRT-KmR-FRT	This study
HK225	BL21(DE3) Δ*adhE*::FRT Δ*pta*::FRT-KmR-FRT	This study
HK126	BL21(DE3) Δ*adhE*::FRT Δ*tpx*::FRT-KmR-FRT, *fnr^+^*	This study
HK128	BL21(DE3) Δ*adhE*::FRT Δ*galM*::FRT-KmR-FRT, *modE^+^*	This study
HK212	BL21(DE3) Δ*adhE*::FRT Δ*ybaV*::FRT-KmR-FRT, *lon^+^*	This study
HK220	BL21(DE3) Δ*adhE*::FRT Δ*ybcH*::FRT-KmR-FRT, *ompT^+^*	This study
HK246	BL21(DE3) Δ*adhE*::FRT Δ*ydbK*::FRT-KmR-FRT, *recE^+^*	This study
HK283	BL21(DE3) Δ*adhE*::FRT Δ*yjjN*::FRT-KmR-FRT, *hsd^K-12^*	This study
HK360	BL21(DE3) Δ*adhE*::FRT Δ*mutL*::FRT-KmR-FRT	This study
HK362	BL21(DE3) Δ*adhE*::FRT Δ*mutS*::FRT-KmR-FRT	This study
HK363	BL21(DE3) Δ*adhE*::FRT Δ*mutT*::FRT-KmR-FRT	This study
**PHAGE**		
P1 *vir*	*vir* mutations	S. Adhya
**PLASMID**		
pCP20	Temperature-sensitive plasmid with an FLP recombinase capable of recognizing the FRT sequence, ApR	CGSC

* Whole genomic sequencing was performed.

Individual gene knockout mutants of *E. coli* in the Keio collection (Baba et al., 2006) were purchased from Open Biosystems (Lafayette, CO). The open reading frames in the individual genes were replaced by kanamycin markers. The mutations were transferred to other strains by standard P1 transduction (Miller, 1992). To transfer the Δ*adhE* mutation, P1 *vir* phage lysates of kanamycin-resistant strain BW25113 Δ*adhE* (JW1228) in the Keio collection were used to transduce the MG1655, W3110, BL21(DE3), REL606, and BL21 strains. If needed, the plasmid pCP20 (Cherepanov and Wackernagel, 1995) was transformed into *E. coli* strains to delete the kanamycin resistance gene from their chromosomes by FLP recombinase at 30°C. Subsequently, plasmid pCP20 with a temperature-sensitive replication origin was cured at 42°C, resulting in the kanamycin-sensitive strains.

To introduce point mutations of the *pta* and *pflB* genes into *E. coli* K-12 Δ*adhE* strains, Δ*ycaN*::KmR and Δ*yfbU*::KmR cassettes obtained from Keio collection were electroporated into mutant cells to tag *pflB*^(G1105A)^ and *pta*^(C2074T)^ mutations, respectively, harboring pKD46 plasmids after lambda recombinase was fully induced by L-arabinose. Subsequently, P1 lysates of the resulting cells were used to transduce kanamycin marker-tagged *pflB*^(G1105A)^ and *pta*^(C2074T)^ mutations into kanamycin-sensitive BW25113 Δ*adhE* backgrounds. In B strain, the same above strategy was employed to individually introduce *pta*^(A1967C)^ and *pflB*^(G1931A)^ mutations into kanamycin-sensitive BL21(DE3) Δ*adhE* (Table 1).

To transfer several authentic genes of K-12 strain to B strain background, we used Pl lysates of JW1317, JW0739, JW0432, JW0556, JW1378, and JW5793 to transduce *fnr^+^, modE^+^, lon^+^, ompT^+^, recE^+^*, and *hsd^K-12^* genes (tagged by adjacent kanamycin resistance genes) into BL21(DE3) Δ*adhE* strain to make HK126, HK128, HK212, HK220, HK246, and HK283 strains. To introduce deletion mutations of Δ*mutL*, Δ*mutS*, and Δ*mutT* genes of Keio collection into BL21(DE3) Δ*adhE* strain, we carried out P1 transduction to make HK360, HK362, and HK363 strains. Other strains were genetically constructed using P1 phage transduction when needed, and also verified by PCR or DNA sequencing (Table 1).

### Anaerobic culture

LB broth and yeast extract were purchased from Becton Dickinson (Sparks, MD). D-glucose, sodium bicarbonate, sodium phosphate monobasic monohydrate, potassium phosphate dibasic, and sodium sulfide nonahydrate were purchased from Sigma-Aldrich (St. Louis, MO). Bacterial seed cultures were grown in 5 mL LB broth at 37°C with shaking at 180 rpm. One milliliter of seed culture was inoculated into a 125 mL serum vial containing 100 mL of fermentation medium, as described previously (Kim et al., 2013). If needed, D-gluconate (final 50 mM), D-fructose, and D-mannitol were respectively added to the fermentation medium as a major carbon source instead of D-glucose. The headspace in serum vials was filled with N_2_ gas and Na_2_S·9H_2_O (final 1 mM) was added to yield strictly anaerobic conditions. Bacterial cells were cultured anaerobically at 37°C with shaking at 180 rpm. Cell growth was monitored by measuring optical density at 600 nm using an Ultrospec 8000 spectrophotometer (GE Healthcare, Uppsala, Sweden). The cell cultures were diluted 1:10 using the same media to measure the optical density accurately.

### Metabolite analysis

The concentrations of metabolites including D-glucose and lactate in the culture were determined by high-performance liquid chromatography (RID-10A RI monitor, Shimadzu, Japan) using an Aminex HPX-87H column (300 × 7.8 mm, Hercules, BioRad) as described previously (Lee et al., 2005). After centrifugation of the cell culture broth, the supernatant was passed through a 0.2 μm syringe filter. The column was isocratically eluted at 47°C with a flow rate of 0.5 mL min^−1^ using 0.01 N H_2_SO_4_. The intracellular concentrations of NAD^+^ and NADH were measured using a NAD^+^/NADH quantification kit (BioVision Inc., Milpitas, CA). Cells were harvested by centrifugation at 10,000 × *g* for 10 min and resuspended in cold phosphate buffered saline solution with an OD_600_ of 1.0. Preparation of NADH extraction buffers and all subsequent steps were performed according to the manufacturer's protocols.

### Genome analysis

The genomic DNAs of *E. coli* strains were purified using the Wizard Genomic DNA purification kit purchased from Promega (Madison, WI). Genome sequences from the parental strains and their derivatives were obtained from an Illumina HiSeq 2000 platform. The 101-cycle paired end reads, with 2.46-3.88 Gb range (30,901,022 reads on average), were produced from 500 bp genomic libraries and processed by the CASAVA 1.9 pipeline. Pretreatment of the reads, reference mapping, and variant detection were carried out using CLC Genomics Workbench version 4.5. Reads shorter than 50 nt were filtered out after quality trimming using a modified Mott algorithm (quality cutoff 0.01), which allowed one or less ambiguous base (N) per read. For reference mapping, the genome sequences of *E. coli* K-12 subst. MG1655 (NC_000913.2) and BL21(DE3) (CP001509.3) were used for K-12 and B lineage, respectively. The default mapping parameters were applied (similarity = 0.8, length fraction = 0.5). Variants in SNP and DIP (deletions, insertions, and polymorphisms) called by the CLC Genomics Workbench were validated to identify mutations that had occurred only in the descendants, but not in the parental strains [BW25113 Δ*adhE* and BL21(DE3) Δ*adhE*]. Nucleotide discrepancies in the *adhE* region owing to P1 transduction, which was used for the disruption of the *adhE* gene, were excluded and later confirmed by comparing *de novo*-assembled contigs. The *pta* and *pflB* genes including promoter regions were amplified by PCR using chromosomal DNAs of *E. coli* parental and derivative strains as templates, and mutations in the corresponding genes were identified by Sanger DNA sequencing.

### Viability test

Both K-12 Δ*adhE* and B Δ*adhE* cells were grown anaerobically in the fermentation medium. All cell cultures (1 ml) were collected from the medium during anaerobic fermentation as mentioned above, and samples diluted with PBS solution were then spread on LB agar plates. After 24 h of aerobic incubation at 37°C, the number of culturable cells was counted for by multiplying the number of colonies on LB agar plates with the corresponding dilution fold. Viability values were obtained by calculating the log base 10 of numbers of culturable cells per 1 ml. For mutant frequency test, Δ*adhE* mutant cells were aerobically grown in LB broth for 12 h at 37°C, diluted with PBS solution, spread on LB agar and incubated aerobically for 24 h at 37°C. The same volumes of Δ*adhE* mutant cells aerobically grown in LB for 12 h at 37°C were spread on the fermentation medium agar and anaerobically incubated using AnaeroGen™ anaerobic pouch system (Oxoid, Hampshire, UK) for 72 h at 37°C.

### Inoculum dilution test

Colonies of K-12 Δ*adhE* and B Δ*adhE* strains, and adapted K-12 Δ*adhE* and B strains Δ*adhE* were singly isolated and inoculated for starter cultures in LB broth. Cell cultures (1 ml) were harvested, resuspended in sterile water (1 ml), serially diluted (1:10), and inoculated in the fermentation medium. Cell growth was monitored by measuring optical densities at 600 nm of culture broths sampled every 3 h.

## Results

### Comparative anaerobic fermentation by Δ*adhE* mutant strains of *e. coli* K-12 and B

**Figure 2:**
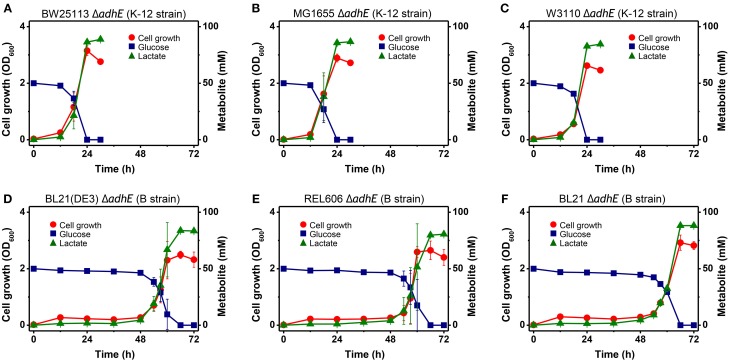
**Anaerobic lactic acid fermentation profiles of various *E. coli* K-12 (A–C) and B (D–F) strains with Δ*adhE* mutations**. **(A)** BW25113 Δ*adhE* (JW1228), **(B)** MG1655 Δ*adhE* (HK104), **(C)** W3110 Δ*adhE* (HK256), **(D)** BL21(DE3) Δ*adhE* (HK110), **(E)** REL606 Δ*adhE* (HK255), and **(F)** BL21 Δ*adhE* (HK105).

Under anaerobic conditions, wild-type *E. coli* K-12 cells (BW25113) completely consumed glucose in 8 h via mixed acid fermentation pathways (Figure 1), which produced succinate, lactate, acetate, ethanol, and formate from D-glucose (Table 2). The BW25113 Δ*adhE* mutant cells (JW1228) mainly produced lactate under anaerobic conditions (Figure 2A). The complete consumption of 50 mM glucose resulted in the production of lactate (>80 mM) by the mutant within 24 h. To verify the effect of the single Δ*adhE* gene mutation on lactic acid fermentation, the Δ*adhE* mutation was introduced into other *E. coli* K-12 (MG1655, and W3110) and B strains [REL606, BL21(DE3), and BL21] via P1 transduction, and anaerobic fermentation was induced. In the *E. coli* MG1655 Δ*adhE* (HK104) and W3110 Δ*adhE* (HK256) strains, the anaerobic lactate fermentations were completed within 24 h, which was similar to the BW25113 Δ*adhE* (JW1228) strain (Figures 2B,C).

**Table 2:** **Anaerobic fermentation profiles of wild-type cells and Δ*adhE* derivatives of *E. coli* K-12 and B strains**.

**Strain**		**Genotype**	**Time (h)^a^**	**OD_600 nm_**	**Specific growth rate (h^−1^)^b^**	**Concentration of substrate or product (mM)**					
						**Glucose^c^**	**Acetate**	**Ethanol**	**Formate**	**Lactate**	**Succinate**
*E. coli* K-12	BW25113	Wild-type	8	5.7 ± 0.2	0.64 ± 0.01	ND^d^	38.9 ± 0.1	40.6 ± 0.1	70.6 ± 0.9	13.5 ± 0.1	6.4 ± 0.1
	JW1228	Δ*adhE*	24	3.4 ± 0.3	0.25 ± 0.04	ND	4.9 ± 0.4	ND	4.8 ± 0.4	86.3 ± 0.2	4.8 ± 0.1
	HK187	Δ*adhE pta*^G1780A^	9	3.4 ± 0.1	0.59 ± 0.01	ND	5.6 ± 0.1	ND	5.7 ± 0.1	87.0 ± 0.9	4.8 ± 0.1
	HK235	Δ*adhE* Δ*pflB*	12	3.1 ± 0.1	0.56 ± 0.02	ND	1.3 ± 0.1	ND	ND	83.2 ± 0.5	2.8 ± 0.1
	HK227	Δ*adhE* Δ*pta*	12	3.1 ± 0.1	0.52 ± 0.01	ND	1.5 ± 0.1	ND	0.7 ± 0.1	79.5 ± 0.6	5.0 ± 0.1
*E. coli* B	BL21(DE3)	Wild-type	8	5.0 ± 0.2	0.67 ± 0.03	ND	41.4 ± 0.1	39.2 ± 0.2	80.5 ± 0.2	13.0 ± 0.1	6.4 ± 0.1
	HK110	Δ*adhE*	72	2.5 ± 0.1	0.17 ± 0.02	ND	4.4 ± 0.3	ND	5.5 ± 0.2	83.7 ± 0.8	4.6 ± 0.5
	HK122	Δ*adhE pta*^A1967C^	15	2.1 ± 0.1	0.31 ± 0.01	ND	4.6 ± 0.1	ND	6.6 ± 0.1	88.0 ± 1.2	4.0 ± 0.1
	HK236	Δ*adhE* Δ*pflB*	15	2.4 ± 0.1	0.27 ± 0.04	ND	1.4 ± 0.1	ND	ND	87.1 ± 0.5	1.2 ± 0.1
	HK225	Δ*adhE* Δ*pta*	24	2.7 ± 0.1	0.35 ± 0.09	ND	2.0 ± 0.2	ND	2.7 ± 0.1	80.9 ± 0.6	3.6 ± 0.1

a Fermentation time (h) when glucose was completely consumed.

b Specific growth rates were measured using exponential phase data.

c Residual glucose concentration. 50 mM glucose was added initially in the fermentation medium.

d ND, not detected.

Unexpectedly, the Δ*adhE* mutants in the B strain background (BL21(DE3), REL606, and BL21) showed long lag periods (approximately 48 h after inoculation), which were followed by exponential growth, rapid glucose consumption, and lactate production (Figures 2D–F). Genotypic and phenotypic verification of the cells was performed by 16S rRNA gene sequencing and single colony isolation on MacConkey agar containing D-galactose or D-lactose before and after anaerobic fermentation to exclude the possibility of additional microbial contamination. The results indicate that the K-12 and B strains lacking alcohol dehydrogenase performed the same lactate fermentation for anaerobic cellular growth. However, the growth speed of *E. coli* Δ*adhE* cells differed in a strain-dependent manner under anaerobic conditions.

### Accelerated anaerobic growth by adapted Δ*adhE* mutant strains

**Figure 3:**
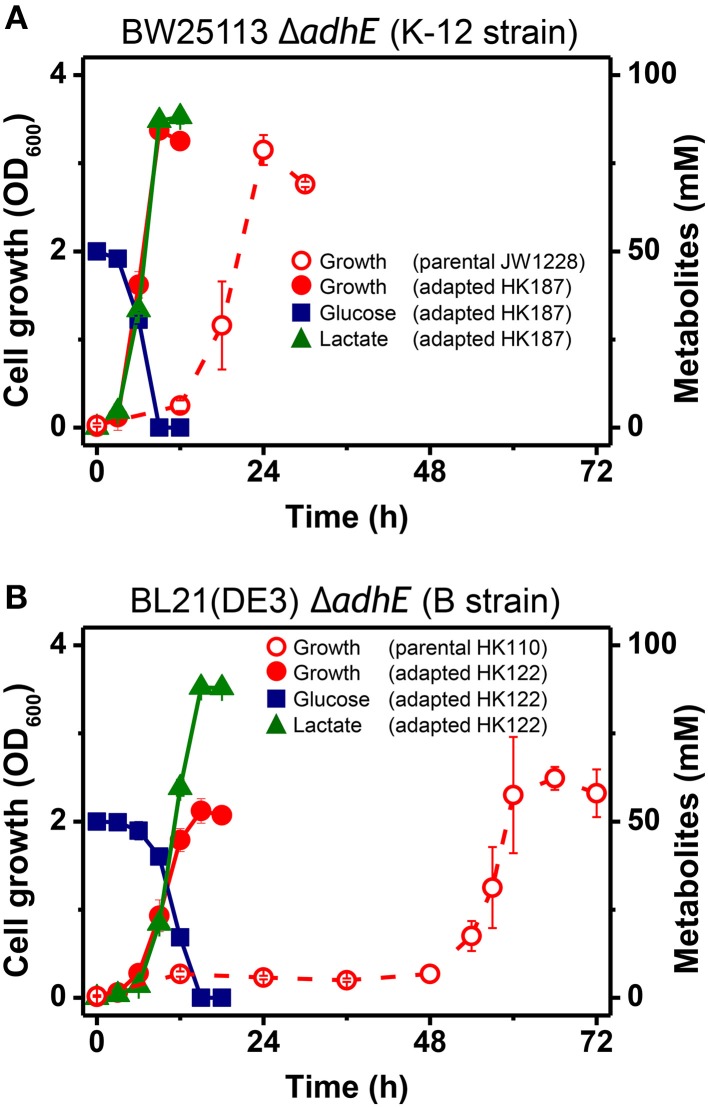
**Rapid anaerobic cellular growth of anaerobically-adapted *E. coli* K-12 Δ*adhE* mutants (A) and B Δ*adhE* mutants (B)**.

During the characterization of the anaerobic lactate fermentation reaction in K-12 Δ*adhE* and B Δ*adhE* strains, we observed that several anaerobic cultures showed unusually rapid growth. For example, the variant strains HK187 and HK122 were obtained as pure isolates from fermentation broths of BW25113 Δ*adhE* (JW1228) and BL21(DE3) Δ*adhE* (HK110), respectively, and then cultured anaerobically in serum bottles. The progeny strain (HK187) completed lactate fermentation within 12 h, whereas anaerobic fermentation by the parental JW1228 cells required 24 h (Figure 3A). Moreover, the adapted progeny cells (HK122) derived from BL21(DE3) Δ*adhE* did not exhibit a long lag period and they completed lactate fermentation within 15 h (Figure 3B), indicating that anaerobic growth by the K-12 and B mutant strains was modified after they underwent anaerobic fermentation. We randomly isolated 37 and 45 additional adapted strains from separate anaerobic cultures of JW1228 and HK110, respectively. Most of the adapted K-12 and B variants produced lactate as a major fermentation product within 12 and 24 h, respectively, which was faster compared to their parental strains (Table 3).

**Table 3:** **Mutation analysis and fermentation profiles of anaerobically-adapted *E. coli* K-12 Δ*adhE* and B Δ*adhE* cells**.

**Strain**	**Gene**	**Mutation type**	**DNA sequence characterization**	**Time (h)^a^**	**OD_600_**	**Concentration of substrate or product (mM)**					
						**Glucose^b^**	**Acetate**	**Ethanol**	**Formate**	**Lactate**	**Succinate**
*E. coli* K-12 BW25113 Δ*adhE* (37)^c^	*pta* (19)	Substitution (12)	T275C (V92A)^d^	12	3.41	ND^e^	5.41	ND	6.20	75.18	3.37
			T389C (L130P)	12	3.43	ND	5.48	ND	6.05	76.17	3.42
			A1546T (T516S)	18	2.69	ND	5.48	ND	6.19	75.84	2.54
			C1576T (Q526Z^f^)	12	3.76	ND	1.15	ND	1.45	81.92	5.31
			G1629C (M543I)	12	3.40	ND	5.70	ND	7.50	92.00	4.00
			G1780A (G594S)	12	3.00	ND	5.60	ND	7.50	92.20	4.50
			C1870A (P624T)	12	2.70	ND	3.60	ND	3.30	91.50	5.90
			A1897T (T663S)	12	3.50	ND	5.12	ND	5.97	83.43	3.62
			A2003C (Q668P)	12	3.72	ND	1.03	ND	1.40	82.06	5.45
			C2074T (R692C)	12	2.90	ND	1.20	ND	1.50	91.90	5.40
			G2078C (G693A; found two times)	12	3.41	ND	5.21	ND	6.05	75.90	3.19
		Deletion (7)	Δ83(G310-A392)	12	2.70	ND	1.10	ND	1.30	92.10	5.60
			G397	12	3.50	ND	1.02	ND	1.46	82.07	5.46
			Δ7(G422-A428)^*^	12	3.52	ND	1.04	ND	1.51	81.60	5.34
			Δ75(C1229-C1303)^g^	12	3.53	ND	1.37	ND	1.57	74.87	4.74
			Δ83(T1786-G1868)	12	3.47	ND	1.32	ND	1.67	76.00	4.59
			Δ13(C1873-G1885)	12	3.50	ND	1.46	ND	1.56	76.39	4.71
			G2031	12	3.80	ND	1.37	ND	1.44	76.28	4.71
	*pflB* (18)	Substitution (10)	A1C (fM1L; loss of start codon)	12	2.50	ND	2.20	ND	ND	99.10	4.50
			C757T (Q253Z)	12	2.40	ND	1.10	ND	ND	96.80	3.80
			A854T (D285V)	12	3.30	ND	1.42	ND	ND	80.75	2.67
			C907T (Q303Z)	12	3.20	ND	1.29	ND	ND	87.34	3.37
			G1105A (E369K)	12	2.40	ND	1.70	ND	ND	96.30	4.10
			C1804T (Q602Z)	12	3.47	ND	1.95	ND	ND	80.24	2.84
			C1862A (P621Q)	12	3.13	ND	2.26	ND	ND	86.44	3.63
			G1898A (G633D)	12	3.57	ND	1.09	ND	ND	82.20	5.58
			A1985C (D662A)	12	3.44	ND	2.26	ND	ND	86.20	3.73
			A2273T (Q758L)	12	3.11	ND	1.38	ND	ND	80.27	2.62
		Deletion (5)	A409 (found three times)	12	2.40	ND	1.50	ND	ND	98.20	4.30
			Δ40(T741-C780)	12	2.95	ND	1.97	ND	ND	80.37	2.58
			Δ11(T1575-G1585)^*^	12	3.13	ND	2.55	ND	ND	80.57	2.62
		Insertion (1)	TGGC after C1463	12	2.98	ND	1.36	ND	ND	80.60	2.72
		IS (2)	IS4 at promoter (−27 nt before AUG codon)	12	2.50	ND	2.00	ND	ND	96.50	4.00
			IS1 after T176	12	2.93	ND	1.31	ND	ND	80.05	2.66
*E. coli* B BL21(DE3) Δ*adhE* (45)	*pta* (42)	Substitution (22)	G265A (E89K)	24	2.21	ND	3.87	ND	4.94	81.93	4.82
			A430C (T144P)	36	2.33	ND	4.89	ND	5.24	74.69	4.28
			T461A (L154Q)	24	2.24	ND	5.40	ND	8.67	88.06	4.60
			A592T (K198Z)	36	2.81	ND	3.15	ND	4.77	88.83	4.84
			G781A (A160T)	24	2.23	ND	5.85	ND	7.73	79.03	4.71
			G895A (E299K)	36	3.40	ND	3.88	ND	5.23	90.61	4.76
			G1011T (W337C; found two times)	24	1.92	ND	4.45	ND	4.92	74.47	4.07
			G1118A (W373Z)	24	2.72	ND	2.02	ND	2.71	78.53	3.20
			C1127A (S376Y)	24	1.65	ND	6.97	ND	8.83	72.80	3.99
			G1523A (G508D)	24	3.00	ND	2.04	ND	2.72	78.00	3.34
			G1535A (G512D)	24	3.02	ND	2.29	ND	2.49	77.30	3.07
			T1634A (L545Q)	24	3.10	ND	6.30	ND	7.50	87.60	4.30
			G1795A (G599S)	24	1.91	ND	5.54	ND	7.07	73.07	4.18
			T1874A (L625Q)	24	2.59	ND	3.40	ND	5.80	91.62	4.33
			T1934C (V645A)	24	2.72	ND	3.08	ND	5.21	92.92	4.03
			A1967C (D656A)	24	1.80	ND	4.60	ND	6.10	80.50	4.70
			A1984T (T662S)	24	1.87	ND	4.50	ND	4.99	75.30	4.20
			C2002T (Q668Z)	24	2.83	ND	2.28	ND	2.50	78.09	3.21
			T2018A (L673Q; found two times)	24	2.14	ND	5.19	ND	7.97	87.13	4.42
			G2077A (G692S)	24	2.21	ND	4.71	ND	6.83	80.15	4.61
		Deletion (14)	C150	24	3.39	ND	1.56	ND	3.16	82.66	3.52
			G219	24	3.26	ND	2.40	ND	3.15	84.29	3.50
			A337	24	3.28	ND	2.29	ND	2.61	77.56	3.03
			G548	36	3.21	ND	3.87	ND	4.94	90.74	4.67
			Δ9(C1198-G1206)	24	2.87	ND	2.22	ND	2.07	76.59	2.90
			Δ75(C1229-C1303)^g^	24	2.97	ND	2.25	ND	2.31	77.18	2.95
			Δ228(A1472-G1699; found two times)	24	3.01	ND	1.88	ND	2.69	77.99	3.29
			Δ135(A1511-G1645)	36	2.72	ND	2.87	ND	3.98	91.07	4.72
			G1598 (found two times)	24	3.01	ND	2.27	ND	2.39	76.21	2.92
			Δ3(G1790-G1792)^*^	24	2.04	ND	4.01	ND	4.86	69.39	3.88
			Δ144(A1802-G1945)	36	2.20	ND	2.43	ND	2.38	76.89	5.02
			Δ13(A1877-C1889)	24	2.26	ND	7.54	ND	9.72	78.41	4.44
		Insertion (5)	8 bp after A212	36	2.90	ND	2.89	ND	4.14	90.31	4.58
			14 bp after A212	24	2.88	ND	2.97	ND	3.32	78.10	3.42
			6 bp after A522	24	2.58	ND	5.66	ND	8.96	88.73	4.12
			23 bp after A1366	24	2.77	ND	2.91	ND	3.52	75.99	2.99
			G after G1897	24	2.80	3.30	2.50	ND	2.13	71.63	2.53
		IS (1)	IS1 after C971	36	1.94	ND	2.02	ND	1.53	77.31	5.07
	*pflB* (3)	Substitution (1)	G1931A (G644D)	24	1.03	ND	2.67	ND	ND	79.34	1.76
		Deletion (1)	Δ27(G59-T85)	24	1.93	ND	1.36	ND	ND	82.83	1.49
		IS (1)	IS1 at promoter (−55 nt before AUG codon)	24	1.53	ND	2.55	ND	ND	86.41	2.26

a Fermentation time (h) when glucose was completely consumed.

b Residual glucose concentration. 50 mM glucose was added initially in the fermentation medium.

c Number of identified mutants is indicated in parenthesis.

d Deduced amino acids are indicated in parenthesis in the substitution.

e ND, not detected.

f Z represents premature translational termination by new stop codons.

g The same mutations were found in both K-12 and B strains.

* Direct repeats were not found in three deletions.

### Genetic analysis of adapted Δ*adhE* mutant cells

To investigate the genetic mechanism that facilitated anaerobic adaptation, seven parental and adapted cells of the K-12 and B strains were analyzed by whole genome sequencing (Table 4). Next generation sequencing showed that, compared with the parental K-12 Δ*adhE* (JW1228) and B Δ*adhE* (HK106) strains, the adapted progeny strains (HK185, HK188, HK121, HK122, and HK201) contained only single mutations in either the *pta* or *pflB* gene; however, there were no other mutations in their genomes (Table 4). Therefore, we further analyzed the nucleotide sequences of the *pta* and *pflB* genes of the adapted progeny strains by Sanger sequencing.

**Table 4:** **Genomic analysis of *E. coli* Δ*adhE* parental and adapted progeny strains**.

**Strain**		**Genotype**	**Reads**	**Bases**	**Reads (trimmed)**	**Bases (trimmed)**	**Avg. length (trimmed)**	**Reads matched**	**% Reads matched**	**Fraction of reference covered**	**Avg. coverage**
BW25113 Δ*adhE*	JW1228	None (parental)	27,415,080	2,768,923,080	24,901,016	2,388,749,991	95.93	24,865,044	99.9	1.00	514.06
	HK185	*pta* (C1870A)	28,307,104	2,859,017,504	25,662,987	2,462,304,965	95.95	25,518,543	99.4	1.00	529.72
	HK188	*pflB*::IS4	24,343,522	2,458,695,722	22,053,723	2,114,460,103	95.88	22,012,693	99.8	1.00	454.82
BL21(DE3) Δ*adhE*	HK106	None (parental)	30,033,896	3,033,423,496	27,532,008	2,650,096,371	96.26	27,470,185	99.8	0.99	579.87
	HK121	*pta* (ΔG219)	36,923,622	3,729,285,822	33,510,481	3,211,950,733	95.85	33,473,669	99.9	0.99	703.70
	HK122	*pta* (A1967C)	38,388,434	3,877,231,834	32,483,523	3,002,388,008	92.43	32,404,329	99.8	0.99	656.89
	HK201	*pta* (T2018A)	30,895,496	3,120,445,096	27,996,337	2,684,345,414	95.88	27,954,846	99.9	0.99	587.87

**Figure 4:**
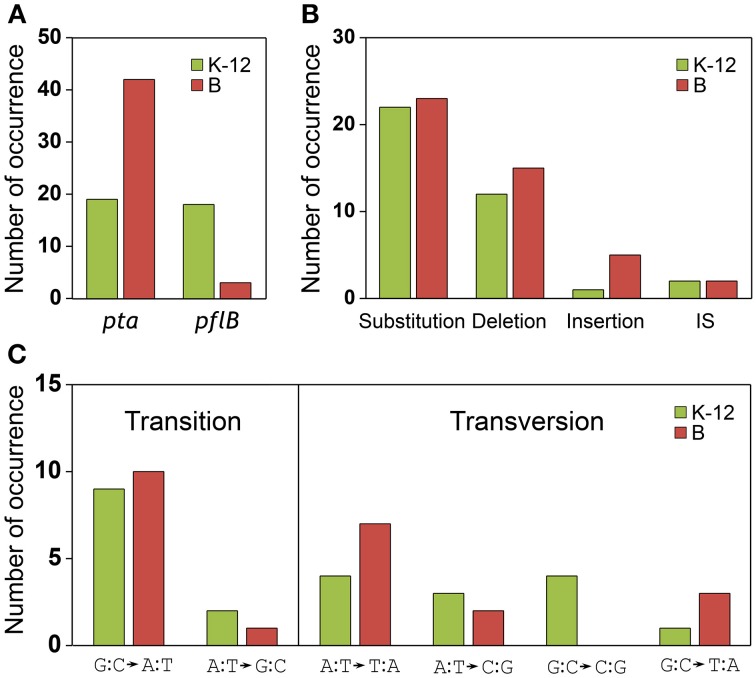
**Analysis of compensatory mutations for anaerobic adaptation in Δ*adhE* mutants of K-12 and B strains**. Thirty seven K-12 mutants (derived from BW25113 Δ*adhE*) and 45 B mutants [derived from BL21(DE3) Δ*adhE*] were analyzed by DNA sequencing. **(A)** Mutation occurrence in *pta* and *pflB* genes, **(B)** types of mutations, and **(C)** types of substitutions were compared in respect of K-12 and B strains.

**Figure 5:**
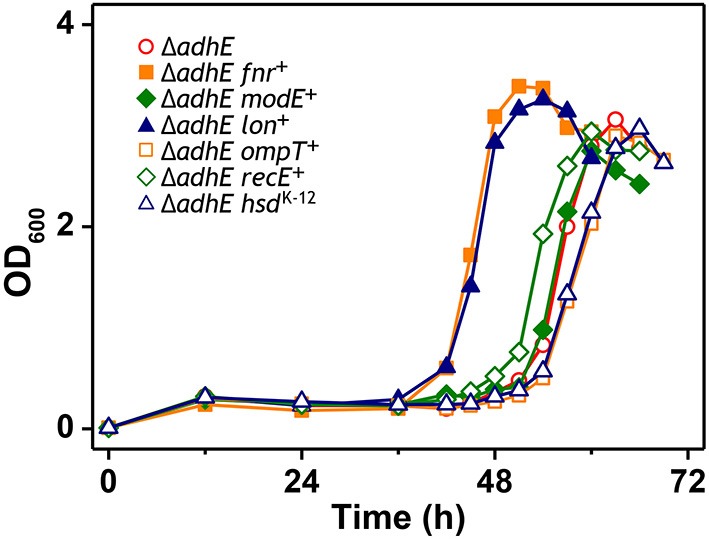
**Anaerobic growth of BL21(DE3) Δ*adhE* cells supplemented with authentic *fnr, modE, lon, ompT, recE*, and *hsd* genes of the K-12 strain**.

The results of the mutational analysis of the adapted K-12 and B strains are presented in Table 3. Of the 37 adapted K-12 *adhE* mutant cells, 19 harbored mutations in the *pta* gene and 18 harbored mutations in the *pflB* gene, including point mutations, deletions, and insertion sequence (IS) elements that caused missense, nonsense, and frame shift mutations. By contrast, the B Δ*adhE* strains exhibited biased mutations in the *pta* gene, where most of the adapted strains (42/45 mutant strains) harbored *pta* mutations, with the exception of three *pflB* mutations (Figure 4A). These results indicate that despite the close relationship between the *E. coli* K-12 and B strains (>99% nucleotide identity) (Jeong et al., 2009), compensatory mutations occurred in preferred target genes under the same anaerobic conditions. Comparative genome studies of K-12 and B strains led us to consider the individual introduction of authentic *fnr, modE, lon, ompT, recE*, and *hsd* genes of the K-12 strain into the BL21(DE3) Δ*adhE* mutant, which would remove its long lag phase (~48 h). As a result, the lag phases (~36 h) of *fnr^+^* and *lon^+^* cells of BL21(DE3) Δ*adhE* backgrounds were not as short as those (~12 h) of the K-12 Δ*adhE* cells during anaerobic adaptation, and those of other strains (*modE^+^, ompT^+^, recE^+^*, and *hsd*^*K*−12^) of BL21(DE3) Δ*adhE* cells were not affected (Figure 5).

**Figure 6:**
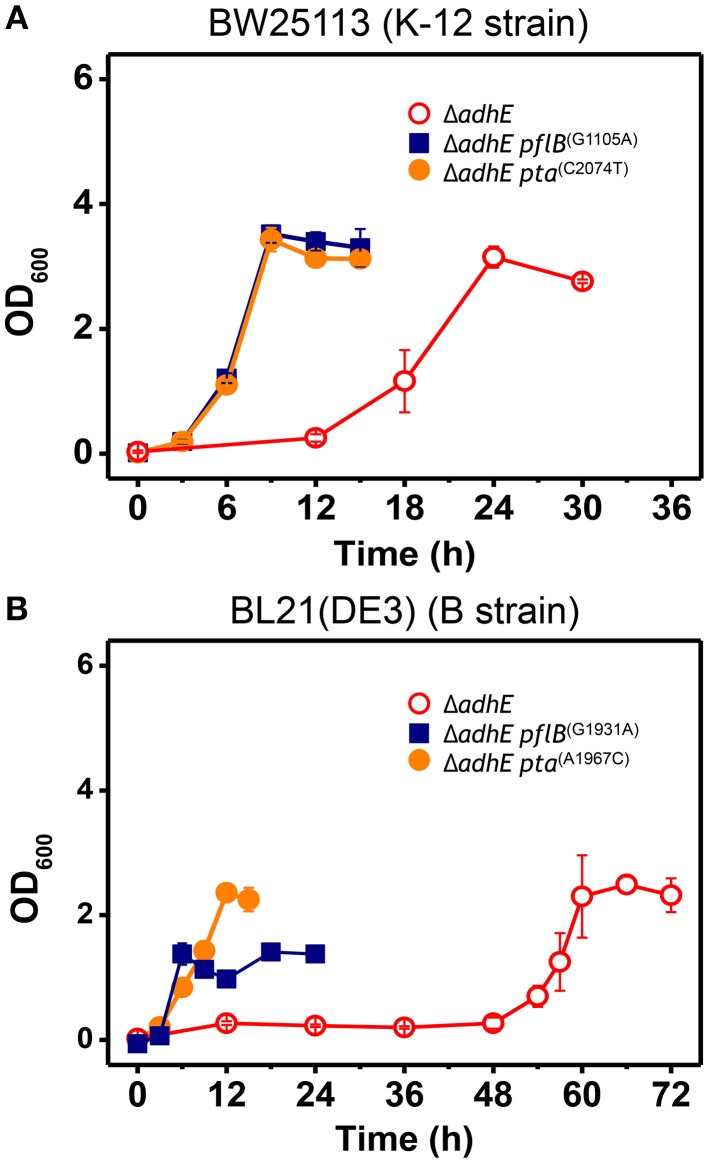
**Accelerated growth of Δ*adhE* mutants of *E. coli* K-12 and B strains by introduction of *pta* or *pflB* point mutations**. **(A)** BW25113 Δ*adhE* cells carrying the *pflB*^(G1105A)^ or *pta*^(C2074T)^ mutation in the genome instead of the authentic *pflB* or *pta* gene, and **(B)** BL21(DE3) Δ*adhE* cells carrying *pflB*^(G1931A)^ or *pta*^(A1967C)^.

To confirm the effects of *pta* and *pflB* mutations on the growth phenotypes in the Δ*adhE* background, we introduced point mutations back into the *pta* and *pflB* genes in the K-12 Δ*adhE* and B Δ*adhE* strains, respectively, and monitored the growth of the mutants relative to that of their parental strains (Figure 6). We found that K-12 Δ*adhE* cells harboring *pta*^(C2074T)^ or *pflB*^(G1105A)^ mutations grew faster than the parental strain and completed fermentation within 12 h. In addition, there were no significant differences in the growth phenotypes of the *pta*^(C2074T)^ and *pflB*^(G1105A)^ mutants in the Δ*adhE* background under anaerobic conditions (Figure 6A). In the B strain background, Δ*adhE pta*^(A1967C)^ and Δ*adhE pflB*^(G1931A)^ cells lacked the long lag periods observed in the Δ*adhE* parental cells and they completed lactate fermentation within 12 and 24 h, respectively (Figure 6B). The Δ*adhE pta*^(A1967C)^ mutants consumed glucose in 12 h and reached a higher final cell density than the Δ*adhE pflB*^(G1931A)^ mutants, which consumed glucose in 24 h. These data showed that the *pta* or *pflB* mutations appeared to result in shorter lag times during the adaptation of Δ*adhE* cells (both K-12 and B strains) to anaerobic conditions.

### Occurrence of various types of mutations during anaerobic adaptation

**Figure 7:**
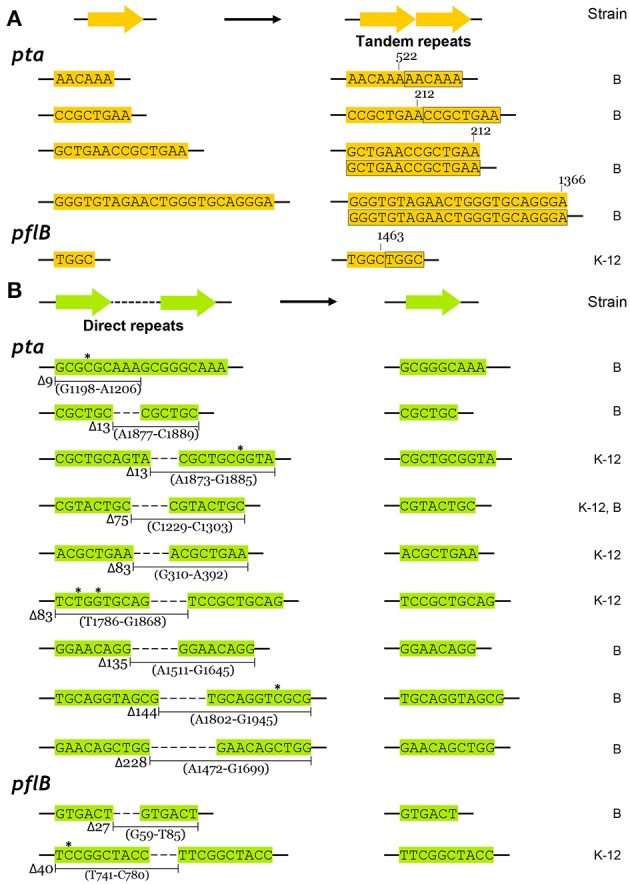
**Repeated sequence mediated insertions and deletions in adapted K-12 and B mutants. (A)** Insertional mutations were generated by precise tandem repeats, and **(B)** deletions between perfect or imperfect direct repeats were observed. Mismatched bases in direct repeats were indicated by asterisks.

We compared the types of mutations that occurred in the adapted K-12 and B strains. In adapted cells, base substitutions were most frequent, which cause nonsense or missense mutations (Figure 4B). Deletions were also frequent, but insertions and IS elements were rarely observed in the adapted strains. Among the base substitutions, G:C to A:T transitions and A:T to T:A transversions were observed frequently in both the K-12 and B strains (Figure 4C). We also found repeat sequences in the insertions and deletions in the *pta* and *pflB* genes (Figure 7). Insertions of tandem repeat sequences (4–23 bp) were confirmed in the K-12 and B strains. Direct repeat-mediated deletions (9–228 bp) were observed frequently in both strains. We found five deletions with imperfect direct repeats (even with 2 bp mismatches out of 10 bp repeats) in the adapted K-12 and B cells.

**Figure 8:**
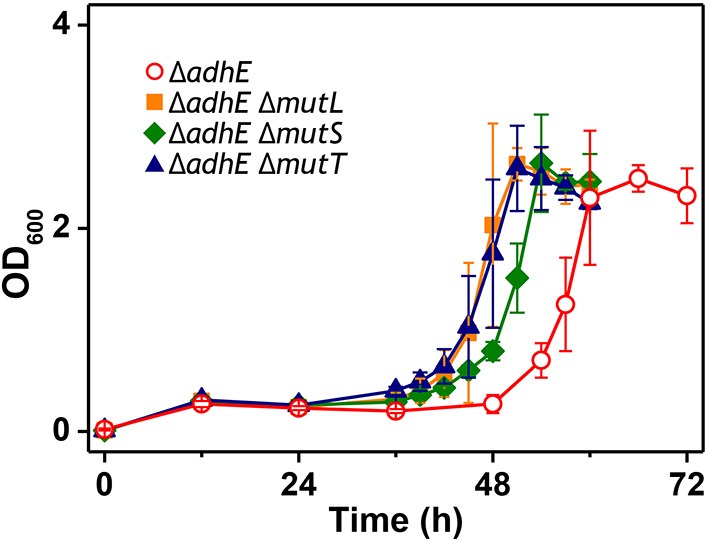
**Effect of mutator gene knockouts on anaerobic adaptation of BL21(DE3) Δ*adhE* cells**.

We observed that the mutant frequency (3.0 ± 0.9 × 10^−6^) in BL21(DE3) Δ*adhE* cells was slightly lower than that (1.2 ± 0.3 × 10^−5^) in BW25113 Δ*adhE* cells. To test whether mutator gene knockouts affect the long lag period of BL21(DE3) Δ*adhE* cells, we introduced three individual mutations of mutator genes (Δ*mutL*, Δ*mutS*, and Δ*mutT*) into BL21(DE3) Δ*adhE* cells and cultured the cells under anaerobic conditions. As a result, the adaptation periods of the B strains were not shortened as those of the K-12 strains (Figure 8).

### Redox cofactor recycling for anaerobic growth

**Figure 9:**
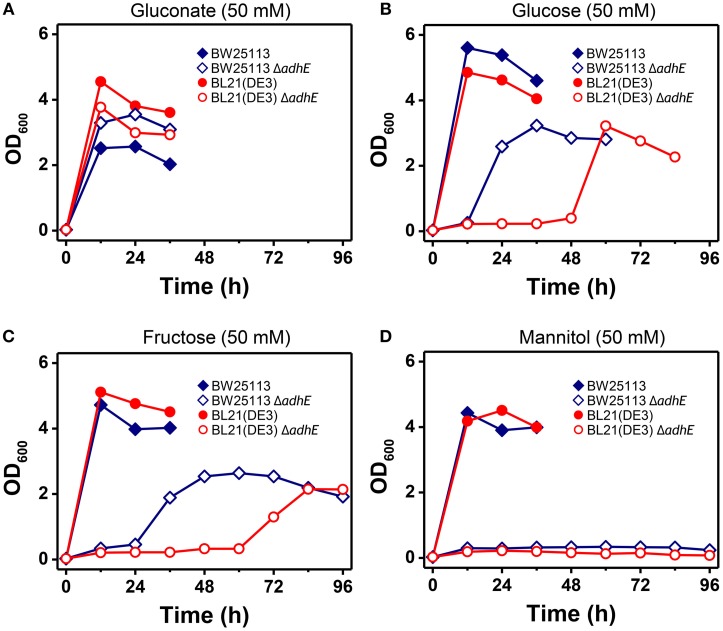
**Anaerobic growth of *E. coli* K-12 Δ*adhE* and B Δ*adhE* cells when various carbon sources with different oxidized states were used**. **(A)** D-Gluconate, **(B)** D-glucose, **(C)** D-fructose, and **(D)** D-mannitol were respectively added to the fermentation medium as a major carbon source.

The phenotypic characteristics of the K-12 Δ*adhE* and B Δ*adhE* cells were investigated by growth on several carbon sources under different reducing conditions (Figure 9). Assuming that *E. coli* cells fully metabolize D-gluconate, D-glucose (or D-fructose), and D-mannitol to pyruvate under anaerobic conditions, the moles of NADH generated would be 1, 2, and 3 (per six carbons), respectively. K-12 and B cells harboring Δ*adhE* mutations did not grow in the presence of reduced D-mannitol, even within 120 h. However, the K-12 Δ*adhE* and B Δ*adhE* cells grew well in a medium that contained oxidized D-gluconate and there was no lag period under anaerobic conditions. These results indicate that Δ*adhE* mutants might experience severe problems with NAD^+^ recycling during anaerobic metabolism. Therefore, we next determined the intracellular levels of NAD^+^/NADH in the K-12 and B strains.

**Figure 10:**
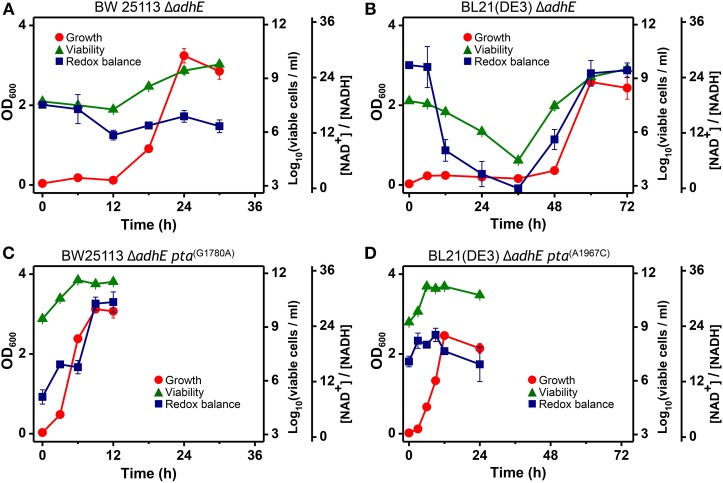
**Analysis of intracellular redox balance and viability of anaerobically grown cells of BW25113 Δ*adhE* (A), BL21(DE3) Δ*adhE* (B), adapted BW25113 Δ*adhE pta*^(G1780A)^ (C), and adapted BL21(DE3) Δ*adhE pta*^(A1967C)^ (D)**.

The intracellular NAD^+^/NADH ratio is a biomarker of metabolic activity (Heikal, 2010). Thus, we measured the viability and redox cofactor ratio [NAD^+^/NADH] of Δ*adhE* mutant cells in the K-12 and B strains to elucidate how cells adapt to anaerobic stress (Figure 10). Under anaerobic conditions, the BW25113 Δ*adhE* cells maintained their intracellular redox balance and viability until 6 h after inoculation into a fermentation medium that contained D-glucose. The viability and redox balance were affected slightly between 6 and 12 h, but recovered after 12 h (Figure 10A). The cellular viability and redox balance of the BL21(DE3) Δ*adhE* cells were markedly affected between 6 and 36 h, but subsequently recovered as cellular growth increased (based on the optical density) (Figure 10B).

In contrast to the optical density, the redox ratio [NAD^+^/NADH] facilitates real-time monitoring of the metabolic state of cells exposed to stress. The NAD^+^/NADH ratios indicated that Δ*adhE* mutant cells underwent severe anaerobic stress, whereas the adapted mutant cells survived. The aerobic-to-anaerobic transition ability and the viability of K-12 cells were superior to those of B cells under anaerobic stress conditions. We analyzed the NAD^+^/NADH ratios of the adapted mutants of both K-12 and B strains, BW25113 Δ*adhE pta*^(G1780A)^ and BL21(DE3) Δ*adhE pta*^(A1967C)^ (Figures 10C,D). The viable cell numbers and the NAD^+^/NADH ratios did not decrease when the adapted mutant cells were grown under anaerobic conditions. These results indicate that the adapted mutants could recycle NAD^+^ efficiently under anaerobic conditions.

**Figure 11:**
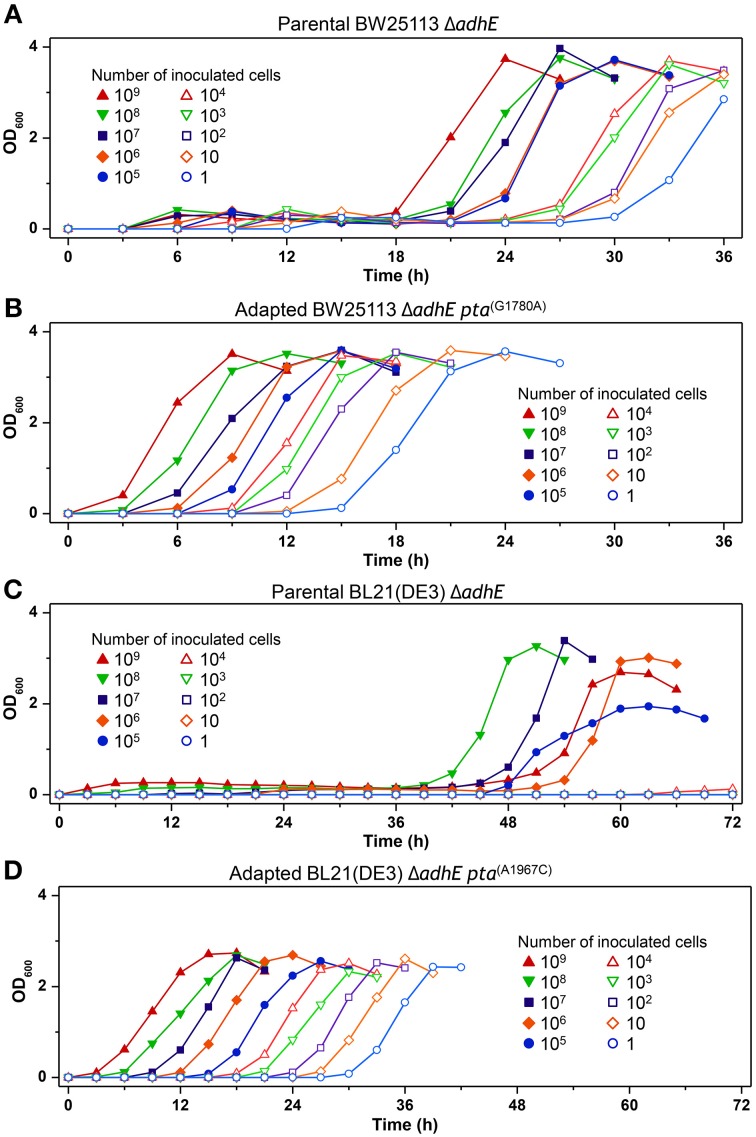
**Inoculum dilution effects on anaerobic growth profiles of parental and adapted cells of BW25113 Δ*adhE* (A), BL21(DE3) Δ*adhE* (B), adapted BW25113 Δ*adhE pta*^(G1780A)^ (C), and adapted BL21(DE3) Δ*adhE pta*^(A1967C)^ (D)**.

We next performed seed dilution experiments to determine the effect of the inoculated cell numbers on adaptation under anaerobic conditions. The results showed that the K-12 Δ*adhE* cells grew and completed lactate fermentation when <10 cells were inoculated into anaerobic culture vials, indicating that these cells could maintain their viability during anaerobic growth (Figure 11A). In the B strain background, smaller inocula of Δ*adhE* cells (10^7^ and 10^8^ cells) grew faster than large inocula (10^9^ cells); this was observed repeatedly and indicated that compensatory mutations occurred randomly during the anaerobic growth of B Δ*adhE* cells, regardless of the inoculum size (Figure 11C). Furthermore, the B Δ*adhE* cells could not adapt and grow within 72 h when <10^5^ cells were inoculated probably because the B Δ*adhE* cells failed to generate a population large enough to acquire spontaneous *pta* or *pflB* mutations. However, the growth speed of the adapted K-12 and B cells appeared to be proportional to the number of inoculated cells (Figures 11B,D).

## Discussion

Routine microbial experiments and even extraordinary achievements in bacterial genetics have begun with the colonies formed on agar plates under certain selectable conditions. When broth cultures start with those colonies, we might assume or wish that the genotypes of inoculated cells are not changed in short period of time (~days). Here we found that the quick adaptive growth of Δ*adhE* cells is accompanied by genomic mutations during anaerobic liquid culture, and also that closely related K-12 and B strains followed different trajectories in adapting to harsh environments.

The genome sequences of K-12 and B cells are very similar (>99% identity) (Jeong et al., 2009), but the main differences between these two strains are the genomic locations of their IS elements, which indicates the important roles of these elements in genomic plasticity and divergence (Schneider et al., 2002). Recently, Yoon et al. determined the differences between *E. coli* B and K-12 strains by performing a comprehensive genome-wide analysis of metabolic networks to identify the genetic bases of their phenotypes via *in silico* complementation testing (Yoon et al., 2012). This analysis showed that *E. coli* B has a greater capacity for amino acid biosynthesis, fewer proteases, lacks flagella, and possesses an additional type II secretion system, whereas *E. coli* K-12 shows higher expression of heat shock genes and is less susceptible to certain stress conditions. In the present study, the K-12 Δ*adhE* strains adapted rapidly to anaerobic stress and grew faster than the B Δ*adhE* strains (Figures 3A,B).

A recent study showed that B strains lack certain anaerobic respiration-related proteins and enzymes, such as H_2_-oxidizing hydrogenases, formate dehydrogenase, nitrate reductase, and the molybdenum-responsive transcriptional regulator (ModE) (Pinske et al., 2011). Given the results of previous comparative genome studies of K-12 and B strains (Jeong et al., 2009; Studier et al., 2009), we anticipated that the individual introduction of authentic *fnr, modE, lon, ompT, recE*, and *hsd* genes of the K-12 strain into the BL21(DE3) Δ*adhE* mutant would abolish its long lag phase. However, we failed to remove the delayed long lag phase of B mutant strains under anaerobic conditions (Figure 5), suggesting that several genes might play concerted roles in the specific anaerobic metabolism processes of K-12 and B strains.

This characterization of adaptation by closely related strains raises intriguing but fundamental questions. First, what is the mechanism responsible for the different compensatory mutations in K-12 and B mutants?

The types of mutations found in *pta* and *pflB* genes were similar, regardless of the strain (Figures 4B,C). Substitutions such as G:C to A:T transitions and A:T to T:A transversions occurred frequently in both strains during adaptation to redox-stress conditions. The insertions were generated by tandem repeat sequences (Figure 7A) and the deletions were mediated by direct repeats (Figure 7B), which might have been caused by slipped-strand mis-pairing during DNA replication and/or homologous recombination; both are regarded as general molecular mechanisms that facilitate bacterial evolution (Albertini et al., 1982; Levinson and Gutman, 1987).

The compensatory mutations that occurred in the *pta* genes K-12 and B strains were found mostly in the C-terminal catalytic domain of the deduced amino acid sequences (Campos-Bermudez et al., 2010) (Table 3). According to the crystal structure of Pta (phosphotransacetylase) from *Methanosarcina thermophile* (Iyer et al., 2004), the *pta* gene mutation in the adapted HK155 strain corresponded to Arg692, which is a proposed putative CoA binding site. Therefore, we assume that *pta* gene mutations might reduce or block further metabolic flux to acetyl phosphate, which can act as a phosphoryl and acetyl donor during cellular signal transduction (Klein et al., 2007; Weinert et al., 2013).

According to the fermentation profiles of adapted mutants (Table 3), it seems that two distinct sets of *pta* mutants exist compared to the metabolite profiles of parental Δ*adhE* cells (Table 2): one with strong reduction of both acetate and formate production such as BW25113 Δ*adhE* cells with C1576T, A2003C, C2074T, or seven individual deletion mutations in *pta* gene, and the other with no significant impact on both acetate and formate production such as the reconstructed HK187and HK122 strains or the adapted BW25113Δ*adhE* cells with the T275C mutation in *pta* gene. Thus, it is likely that some *pta* mutations may restore anaerobic growth while not affecting the formation of acetate and formate, indicating some Pta enzymes in those adapted cells may not be fully inactivated. Moreover, this dichotomy within *pta* mutants is more obvious in the K-12 progeny than in the B derivatives.

In case of adapted mutants via *pflB* gene mutations, all *pflB* mutants could not produce formate, one of reaction products of the PflB enzyme, and exhibited reduced acetate formation (Table 3) as observed for Δ*adhE* Δ*pflB* cells (Table 2), arguing for the inactivation of PflB enzymes in those adapted mutants. It is not clear why compensatory *pflB* gene mutations occurred frequently in K-12 Δ*adhE* strains but rarely in B Δ*adhE* strains. As shown in Figure 6, the growth of the Δ*adhE pflB*^(G1931A)^ mutants was poorer than that of the Δ*adhE pta*^(A1967C)^ mutants in the BL21 (DE3) background, which might explain why we detected fewer *pflB* mutations among the total range of compensatory mutations.

The second question is why did these strains adapt differently in stress conditions?

As mentioned above, we tested whether adaptation could be accelerated by mutator gene knockouts (Δ*mutL*, Δ*mutS*, and Δ*mutT*). It is reported that *mutT* mutations enhance the point mutation rate by 150-fold (Wielgoss et al., 2013); however, these mutations could not shorten the delayed lag phase of the B strains compared to the growth of K-12 strains (Figure 8). These results demonstrate that the mutation rate is not the main explanation for the delayed adaptation of B strains compared with K-12 strains under anaerobic conditions.

The redox cofactor balance [NAD^+^/NADH] in the K-12 Δ*adhE* strains was affected slightly under anaerobic conditions, whereas that of the B Δ*adhE* cells was affected markedly (Figure 10). The recovery of the redox balance [NAD^+^/NADH] in adapted K-12 Δ*adhE* cells was even higher than that in the parental K-12 Δ*adhE* cells (Figure 10C). This indicates that alternative redox balance metabolic pathways might be available in K-12 Δ*adhE* cells. Figure 10 shows that the redox balance was significantly associated with the viability of stressed cells. Thus, it is probable that the culturable cell number of B Δ*adhE* strains is critical for the generation of adapted cells harboring compensatory mutations under severely stressful conditions, which may explain why the B Δ*adhE* strains exhibited delayed adaptive growth compared with the K-12 Δ*adhE* strains. This hypothesis was also strongly supported by the inoculum dilution experiments, which showed that the adaptation speed depended on the number of cells inoculated (Figure 11). We assume that severely stressed B Δ*adhE* cells lost their viability and could not restore cellular growth via adaptations. Under the same anaerobic conditions, K-12 cells may adapt rapidly by altering their metabolic fluxes via alternative and/or bypass reactions to restore their redox balance, because [NAD^+^/NADH] was not severely affected by the absence of AdhE (Figure 10). The present study clearly demonstrated that the requirements for short and long lag periods during microbial adaptation depended mainly on the fraction of metabolically active cells present when bacteria were exposed to adverse conditions.

## Conclusion

Our study identified an unusual short-term microbial adaptation to stressful conditions, which was accompanied by genomic mutations that have been observed frequently during other examples of molecular evolution. We found that even closely related strains possess distinct metabolic characteristics that allow them to adapt in different ways to the same environmental conditions. In biotechnological aspects, engineered bacterial strains may rapidly evolve to cope with adverse metabolic fluxes, which might have been overlooked during the development and fermentation of industrial strains. These events may occur without our knowledge in microbial experiments in the laboratory as well as microbial outbreaks in nature.

### Conflict of interest statement

The authors declare that the research was conducted in the absence of any commercial or financial relationships that could be construed as a potential conflict of interest.
